# Anoikis Resistance as a Further Trait of Acidic-Adapted Melanoma Cells

**DOI:** 10.1155/2019/8340926

**Published:** 2019-06-02

**Authors:** Silvia Peppicelli, Jessica Ruzzolini, Francesca Bianchini, Elena Andreucci, Chiara Nediani, Anna Laurenzana, Francesca Margheri, Gabriella Fibbi, Lido Calorini

**Affiliations:** ^1^Department of Experimental and Clinical Biomedical Sciences “Mario Serio,” Section of Experimental Pathology and Oncology, Italy; ^2^Istituto Toscano Tumori and Center of Excellence for Research, Transfer, and High Education (DENOTHE), University of Florence, Florence, Italy

## Abstract

Melanoma is characterized by a low extracellular pH, which contributes to the development of an aggressive phenotype characterized by several properties as the switch to an epithelial-to-mesenchymal program, the increase of apoptotic resistance, and the migratory ability together with the development of drug resistance. Here, we demonstrate that melanoma cells grown in low pH medium (pH 6.7) for a short (24 hours) or long (at least 3 months) period equally express an anoikis resistance profile. Anoikis is a form of apoptosis prompted by loss of adhesion, particularly requested by aggressive cancer cells to metastasize. Anoikis resistance was ascertained in acidic melanoma cells either grown in agarose-coated plates or incubated in rocking conditions. Both analyses indicate that acidic cells were more able to survive in a nonadherent condition than cells grown in standard pH, an effect resulting in a more cloning efficiency and migratory ability. Ability to survive during rocking was inhibited using mTOR/NF-kB inhibitors. Finally, we checked whether characteristics related to the* in vitro* anoikis resistance acquired by acidic melanoma cells might be also suitable for* in vivo* challenge. We injected acidic melanoma cells into blood stream, and then we verify how many cells survived in blood after 15 min from the injection. Only acidic cells, transient and chronic, survived, whereas melanoma cells grown in standard pH medium did not. Overall, we have had the opportunity to demonstrate that low extracellular pH represents an additional mechanism able to promote an anoikis resistance in solid tumors.

## 1. Introduction

Metastatic disease is a fatal consequence for tumor-bearing patients and circulating tumor cells (CTCs) are the essential precondition for metastasis to occur. CTCs leave the primary tumor, travel through the body's vasculature, and arrest into target organ, extravasating and serving as a seed for the generation of a secondary lesion [1]. In circulation, CTCs are exposed to several critical conditions, such as survival in suspension, shear stress, and immune attack; thus survival can be highly variable disclosing cell populations expressing a “perfect” circulating phenotype [2]. 

Among the several aspects characterizing the circulator phenotype, one of the most critical is anoikis resistance. Anoikis (i.e., “without a house”) was first described in the early 1994 by S. Frish and H. Francis [3] and refers to a form of programmed cell death that occurs when cells detach from their extracellular matrix (ECM). Normal cells of a tissue die during this process in view of their stringent requirement of ECM attachment, whereas certain subpopulations of tumor cells are able to survive also in total absence or inappropriate ECM adhesion. Indeed, cancer cells need to survive after detachment from their primary site and during the travel through the lymphatic and circulatory systems. This means that migratory tumor cells have to acquire anoikis resistance to complete the metastatic cascade; thus resistance to anoikis might be considered a hallmark of metastatic cancer cells [4, 5]. Anoikis is controlled by activation of the mitochondrial apoptotic pathway involving subfamilies of B-cell lymphoma (Bcl)-2 proteins that lead to the activation of the caspase enzymes or is induced by the stimulation of death receptors members of TNF superfamily [6, 7]. Acquisition of anoikis resistance is obtained through different strategies such as stimulation of survival signals (PI3K/Akt, MEK, ERK, and NFkB), inhibition of apoptotic pathways, undergoing EMT, and/or changing the pattern of integrin expression by adapting to the metastatic site [7].

Among the different characteristics of tumor microenvironment we focused on acidosis. Generation of extracellular acidosis is almost an unavoidable phenomenon during tumor cell proliferation. Indeed, proliferating cancer cells located in the proximity of vasculature, where oxygen tension might be enough to sustain an oxidative phosphorylation, exhibit a preferred glycolysis pathway (the so-called “Warburg effect” or aerobic glycolysis), releasing lactate and protons in the external medium [8–10]. When oxygen tension reduces, the stabilization of the hypoxia-inducible (HIF)-1*α* transcription factor drives an anaerobic type of glycolysis leading to an even more pronounced lactate dehydrogenase (LDH)-A-dependent lactate and proton production. Hypoxic cancer cells use the monocarboxylate transporter (MCT)-4 and sodium-proton exporters to discard lactate and protons [11]. The increased aerobic and anaerobic glycolysis pathway may be visualized in tumor-bearing patients using 18F-deoxyglucose positron emission tomography imaging [12]. Overall, most tumor regions experience acidosis (ranging pH 6.7) possibly for a variable period of time, also considering the reduced movement fluids, due to the low lymphatic vessels and the high interstitial pressure in the central areas of tumors [13, 14]. It was demonstrated by our laboratory and others that decreased extracellular pH (pHe) may contribute to the promotion of cancer cells aggressiveness via stimulation of increased mutation rate [15], angiogenic [16] and lymphangiogenic growth factor release [17], metalloprotease-dependent invasiveness into host tissues [18], and metastatic potential [19]. All these characteristics are associated with the acquisition by acidic cancer cells of an epithelial-to-mesenchymal (EMT) phenotype, endowed with a reduced rate of proliferation and a high resistance to apoptosis [20]. Both, quiescence and apoptosis resistance of acidic cancer cells, make these cells a further niche of radio and chemotherapy resistance and evasion from cytotoxic lymphocytes and natural killer cells [14]. Most of changes described in acidic cancer cells are closely associated with NF-kB induction, as mesenchymal phenotype, apoptosis resistance, and oxidative phosphorylation [20, 21], while HIF-1*α* is drastically inhibited by acidosis [22].

All these considerations prompt us to investigate whether acidosis might also promote in cancer cells an anoikis resistance profile, as a part of their migratory phenotype. Here, we demonstrate that transient and chronic acidic-adapted melanoma cells express an anoikis resistance as a new additional trait, which may be targeted by mammalian target of rapamycin (mTOR)/nuclear factor kappa-light-chain-enhancer of activated B cells (NF-kB) inhibitors. These findings add new possible mechanisms to control metastatic spread of melanoma cells.

## 2. Materials and Methods

### 2.1. Cell Lines and Culture Conditions

In this study, we used the melanoma cell line A375M6, isolated in our laboratory as described in our previous work [23]. In some experiments we also used the M21 (kindly provided by Dr. Antony Montgomery, The Scripps Research Institute, La Jolla, CA) and the WM266-4 (from ATCC) human melanoma cell lines. Melanoma cells were cultivated in Dulbecco's Modified Eagle Medium high glucose (DMEM 4500, EuroClone, MI, Italy) supplemented with 10% foetal bovine serum (FBS, Boehringer Mannheim, Germany), at 37°C in humidified atmosphere containing 90% air and 10% CO_2_, as described in our previous work [23].

Cells were harvested from subconfluent cultures by incubation with a trypsin-EDTA solution (EuroClone, MI, Italy) and propagated every three days. Viability of the cells was determined by Trypan blue exclusion test. Cultures were periodically monitored for mycoplasma contamination using Chen's fluorochrome test.

### 2.2. Acidic Treatment

Chemical acidified medium was obtained by the addition of HCl 1N in DMEM4500 10% FBS, following the protocol described in [23]; pH value was monitored by a pH meter (Orion PH Meter 520A-1) and when pH value was stable (6.7±0.1), acidified medium was added to cell cultures and the seal cap was tightly closed. pH was evaluated also at the end of each experiment. For transient acidosis, cells were grown in acidic medium for 24h. For chronic acidosis, melanoma cells were cultured in pH 6.7 medium for 3 months, until tumor cells recovered a similar growth rate as parent cells maintained at pH 7.4. During the long lasting acidic treatment, no significant death of cells was found.

### 2.3. Anoikis Assay

In order to simulate anchorage-independent growth conditions, we performed two different tests culturing melanoma cells in dishes coated with agarose or rocking the cells in tubes.

Culture dishes were coated with 1.5 % agarose (Promega, San Luis Obispo, California).

5x10^4^ cells were plated in agarose-coated petri dishes in complete medium and after 5 days colonies were counted. At the end of the experiments, cells from colonies were centrifuged and used for western blot analysis, invasion assay, and Wound Healing Assay.

In rocking test, 5x10^4^ cells were left rocking in tubes on the Mini Rocker Shaker (Biosan), at room temperature in Dulbecco's D-Mem Nutrient mix F12 (DME/F12-HEPES EuroClone, MI, Italy). In some experiment cells were treated with esomeprazole (Astra Zeneca, Sweden) and everolimus (MedChemExpress, Stockholm Sweden) during the rocking period. After 48h of rocking, cells were centrifuged, counted, and used for western blot analysis, invasion assay, and Wound Healing Assay.

### 2.4. Western Blotting Analysis

Cells were washed with ice cold PBS containing 1 mM Na_4_VO_3_ and lysed in 100 *μ*l of cell RIPA lysis buffer (Merck Millipore, Vimodrone, MI, Italy) containing PMSF (Sigma-Aldrich), sodium orthovanadate (Sigma-Aldrich), and protease inhibitor cocktail (Calbiochem). Immunoblot was performed as reported in [21]. Aliquots of supernatants containing equal amounts of protein in Laemmli buffer were separated on Bolt® Bis-Tris Plus gels 4-12% precast polyacrylamide gels (Life Technologies, Monza, Italy). Fractionated proteins were transferred from the gel to a PVDF nitrocellulose membrane using an electroblotting apparatus (Bio-Rad, Segrate, MI, Italy). Blots were stained with Ponceau red to ensure equal loading and complete transfer of proteins; then they were blocked for 1 hour, at room temperature, with Odyssey blocking buffer (Dasit Science, Cornaredo, MI, Italy). Subsequently, the membrane was probed at 4°C overnight with primary antibodies diluted in a solution of 1:1 Odyssey blocking buffer/T-PBS buffer. The primary antibodies were as follows: rabbit anti-EGFR (1:500 Cell Signaling Technology, Danvers, MA, USA), rabbit anti-N-Cadherin (1:1000 Biorbyt, Cambridge, UK), mouse anti-cleaved PARP 1 (1:200 Santa Cruz Biotechnology, Santa Cruz, California), rabbit anti-p-AKT (Cell Signaling Technology, Danvers, MA, US), rabbit anti-AKT (1:1000 Cell Signaling Technology, Danvers, MA, USA), rabbit anti-pERK (1:1000 Cell Signaling Technology, Danvers, MA, USA), rabbit anti-ERK (1:1000 Cell Signaling Technology, Danvers, MA, USA), mouse anti-tie1 (1:200 Santa Cruz Biotechnology, Santa Cruz, California), mouse anti-IKB alpha (1:500 GeneTex, Irvine, CA USA), and mouse anti-*β* actin monoclonal antibody (1:2000, GeneTex, Irvine, CA,USA). The membrane was washed in T-PBS buffer, incubated for 1 hour at room temperature with goat anti-rabbit IgG Alexa Fluor 680 antibodies (Invitrogen, Monza, Italy), and then visualized by an Odyssey Infrared Imaging System (LI-COR® Bioscience). Mouse anti-*β*-tubulin monoclonal antibody (Sigma, Saint Louis, MO, USA) was used to assess equal amount of protein loaded in each lane.

### 2.5. Annexin V/PI Flow Cytometer Analysis

Apoptosis was measured by flow cytometry, using the Annexin V staining as previously described [23]. Cells incubated in anchorage independent conditions were collected, washed once with PBS, resuspended in 100 *μ*L of 1x Annexin-binding buffer at the concentration of 1 x 10^6^ cells/mL, stained with 5 *μ*L of Annexin V FITC-conjugated (ImmunoTools, Friesoythe, Germany) and 1 *μ*L of 100 *μ*g/ml PI working solution, and incubated at 4°C in the dark condition for 15 min. Then, 400 *μ*L of 1X Annexin Binding Buffer was added to each sample and cells were analyzed by flow cytometry (BD-FACS Canto) to find out the viability (annexin V and PI negative, Q3), early apoptosis (annexin V positive and PI negative, Q4), or late apoptosis (annexin V and PI positive, Q2). A minimum of 10000 events were collected.

### 2.6. Invasion Assay

Invasiveness of A375M6 melanoma cells incubated in anchorage independent conditions was determined* in vitro* on Matrigel (BD Biosciences) precoated polycarbonate filters, with 8 *μ*m pore size, 6.5 mm diameter, 12.5 *μ*g Matrigel/filter, mounted in Boyden's chambers, as previously described [24]. 1,5x10^5^ cells (200 *μ*L) were seeded in their growth medium in the upper compartment and incubated for 6 hours at 37°C in 10% CO_2_ in air. In the lower chamber, complete medium was added as chemoattractant. After incubation, the inserts were removed and the noninvading cells on the upper surface were wiped off mechanically with a cotton swab and the membranes were fixed overnight in ice-cold methanol. Cells on the lower side of the membranes were then stained using the Diff-Quick kit (BD Biosciences) and photographs of randomly chosen fields are taken.

### 2.7. Wound Healing Assay

Cell migration was evaluated by an* in vitro* wound healing assay. The cell layer was wounded with a sterile 200 ml pipette tip and incubated in 1% FBS culture medium for 24 hours. The wound was observed after 24 hours and photographed using phase contrast microscopy.

### 2.8. Cytofluorimetric Assay

The expression of integrin receptors *α*v*β*3 and *α*v*β*5 and MHC class I antigens in melanoma cells was determined by cytofluorimetric analysis (FACScan, Becton Dickinson, USA). 1x10^6^ melanoma cells were incubated for 30 min at 4°C with appropriate primary antibody. Mouse anti-human integrin receptor *α*v*β*3 (clone LM609, Millipore) and mouse anti-human integrin receptor *α*v*β*5 (sc13588, Sigma), mouse monoclonal AF6-88.5.3 specific for class I H-2Kb antigen (provided by Dr. S. Gattoni-Celli, Medical University of South Carolina, USA) (Calorini 1999) or mouse monoclonal HLA-ABC antigen clone W6/32 (DAKO), were used. Cells were washed twice with PBS and then incubated with FITC-conjugated goat anti-mouse Ig (Sigma) for 30 min at 4°C. Cells were washed twice with PBS, fixed in 2% paraformaldehyde, and stored at 4°C in the dark until FACS analysis.

### 2.9. Blood Stream Survival

Investigation has been conducted in accordance with national guidelines and has been approved by the ethics committee of the Animal Welfare Office of the Italian Work Ministry and conformed to the legal mandates and Italian guidelines for the care and maintenance of laboratory animals.

Melanoma cells (1x10^6^ cells) adapted to an acidic medium, either transiently or chronically, and melanoma cells grown under standard conditions (pH 7.4) were inoculated intravenously into SCID/bg immune deficient mice (Aut Min 401/2015-2019). Mice were anesthetized using Isoflurane delivered via a precision vaporizer with a nose cone. 15 min after tumor cells injection, 0,5 ml blood sample was collected through a cardiac puncture using a preheparinized 25 gauge x 1^″^ needle syringe. Red blood cells were lysed mixing nine parts sterile H_2_O for 15 sec and then adding one part 10x PBS. The heterogeneous cell suspensions were distributed in at least 3 culture dishes. The number of colonies was counted after 3 weeks of growth in standard conditions and reported as mean ± SD. Colonies were amplified and pooled together.

### 2.10. Adhesion to Endothelial Cells

Human umbilical vein endothelial cells (HUVECs) were grown to confluence in 24-gelatin precoated well culture plates in complete EGM-2 Bullet Kit (CC-3162 Lonza) with 10% Fetal Bovine serum (FBS) (HyClone) and preactivated with rhIL1b (2ng/ml) for 6h. Subconfluent cultures of tumor cells were rinsed with PBS and exposed to 5*μ*M CellTrace™ CFSE dye for 15 min at 37°C. After incubation cells were incubated in standard conditions 1h, next tumor cells were detached and seeded on endothelial cell monolayers at a 1:1 ratio. Cocultures were incubated at 37°C for 30 min or 60 min. At designated times cocultures were rinsed 3 times with serum free medium to remove nonadherent cells and cells were detached, fixed in 5% paraformaldehyde, and analyzed by flow cytometry. Human melanoma cells were identified as CFSE positive cells and evaluated as percentage of total cell population.

### 2.11. Statistical Analysis

Densitometric data are expressed as means ± standard errors of the mean (SEM) depicted by vertical bars from representative experiment of at least three independent experiments. Statistical analysis of the data was performed by Student's t-test.

## 3. Results

### 3.1. Characteristics of Transient and Chronic Acidic-Adapted Human Melanoma Cells Correlated with an Anoikis-Resistant Phenotype

To analyze the influence of a reduced pH on human melanoma cells correlated with an anoikis resistant phenotype, we exposed A375M6 melanoma cells to a pH 6.7 acidified medium. Since in tumor microenvironment extracellular acidosis could occur as a short-time insult or as a prolonged stressed condition, we used 24h acidified melanoma cells, serving as a model of transient acidosis, and three months acidified A375M6 to evaluate the effect of a prolonged acidosis condition (chronic exposure). In general, chronic exposure in acidosis was considered concluded, when melanoma cells after a period of a reduced cell proliferation regained their original level of proliferative activity. Extracellular pH of melanoma frequently ranges between 6.7 and 6.9; the exposure to an acidic medium did not change viability of melanoma cells although it stopped proliferation during the initial phase of treatment.

**Figure 1:**
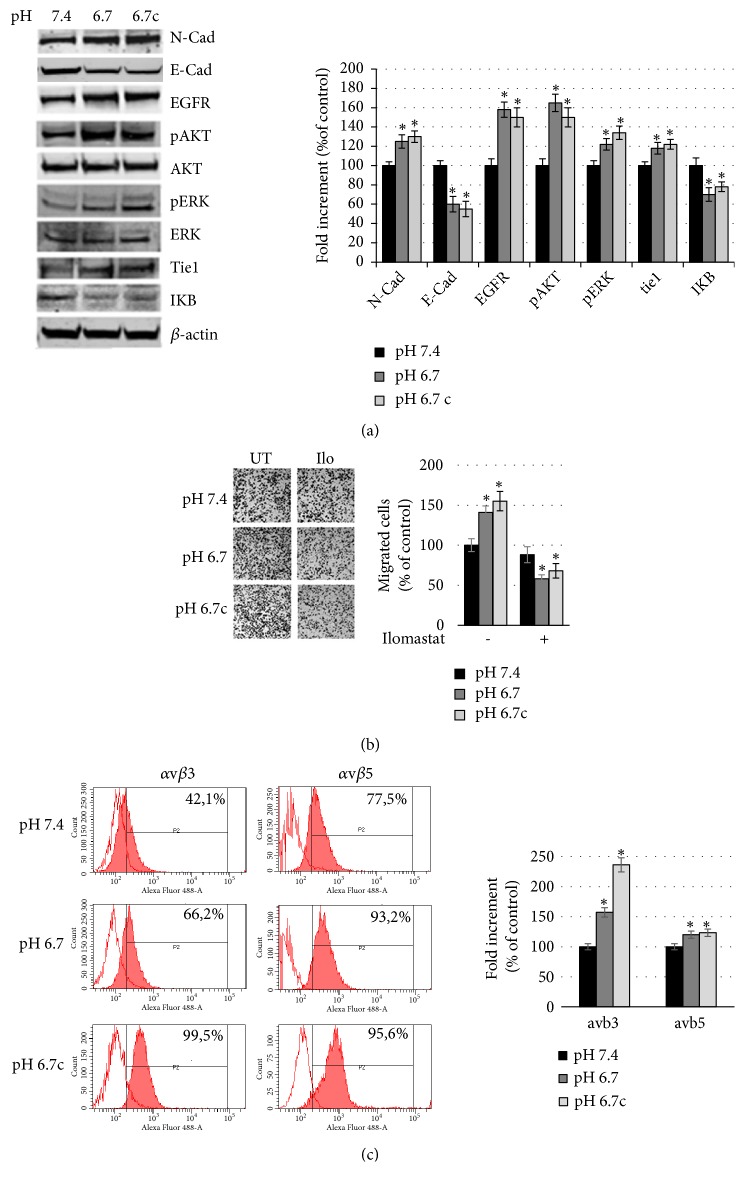
*An anoikis resistant-like phenotype in acidic melanoma cells.* (a) Representative images of western blot for N-cad, EGFR, pAKT/AKT, pERK/ERK, Tie1, IKB, and *β*-actin of A375M6 melanoma cells exposed to standard medium (pH 7.4), to an acidified medium for 24 hours (transient exposure, pH 6.7) or to a reduced pH medium, for approximately three months (chronic exposure, pH 6.7c), and (right) densitometry graph of protein expression. (b) Representative images (left) of invasiveness of melanoma cells grown in different pH conditions in the presence or absence of 25*μ*M Ilomastat (a metalloproteinases inhibitor) and quantitative analysis (right) of the number of cells that migrated through Matrigel. (c) Representative images (left) of flow cytometric analysis of *α*v*β*3 and of *α*v*β*5 integrin expression of A375M6 melanoma cells grown in different pH conditions and (right) quantitative analysis of integrin expression as percentage of increment in Mean Fluorescent Intensity. Each experiment was conducted in triplicate and data are expressed as mean ± SEM of at least three independent experiments. *∗* p<0.05.

As we have already demonstrated, acidic cells, both transient and chronic, express a spindle and dendritic shape concomitant with an upregulation of N-cadherin and a downregulation of E-cadherin (Figure 1(a)), characteristics for an epithelial-to-mesenchymal transition (EMT) program [20, 23].

As other markers of a possible anoikis resistant phenotype, we show (Figure 1(a)) upregulation in acidic melanoma cells of the epidermal growth factor receptor (EGFR), an additional prosurvival signaling mediator linked to ERK and AKT pathways, and of the Tyrosine kinase with immunoglobulin-like and EGF-like domains (Tie)-1, a new class of receptor tyrosine kinases specifically expressed during vascular endothelial cell growth and differentiation. EGFR, Tie-1, and Tie-2 receptors on endothelial cells, once activated, transmit signals through downstream Raf/mitogen-activated protein kinase (MEK)/ERK pathway promoting endothelial cell proliferation and migration which are essential for angiogenesis induction [25].

Recent studies have shown that it is only after the cancer cells resist anoikis that they attain the potential to metastasize [26]. Thus, invasion, a critical step in metastasis, was investigated. We observed a higher ability of acidic cells to invade Matrigel filter (see Figure 1(b)), and this invasive ability was metalloproteinase dependent, as proved by the inhibition of migration using Ilomastat, an MMP inhibitor (Figure 1(b)). Further, in our laboratory we found that these adapted cells were more resistant to proapoptotic agents such as oxygen peroxide and etoposide, a DNA poison for cancer cells [20, 23].

An anoikis resistant phenotype is always correlated with changes in the integrin expression profile [6, 7]. In fact, Figure 1(c) shows that both transient but in particular chronic acidic melanoma cells express a more elevated level of *α*v*β*3 and *α*v*β*5 integrins.

### 3.2. Anoikis Resistant Phenotype in Cells Grown in Agarose-Coated Dishes

**Figure 2:**
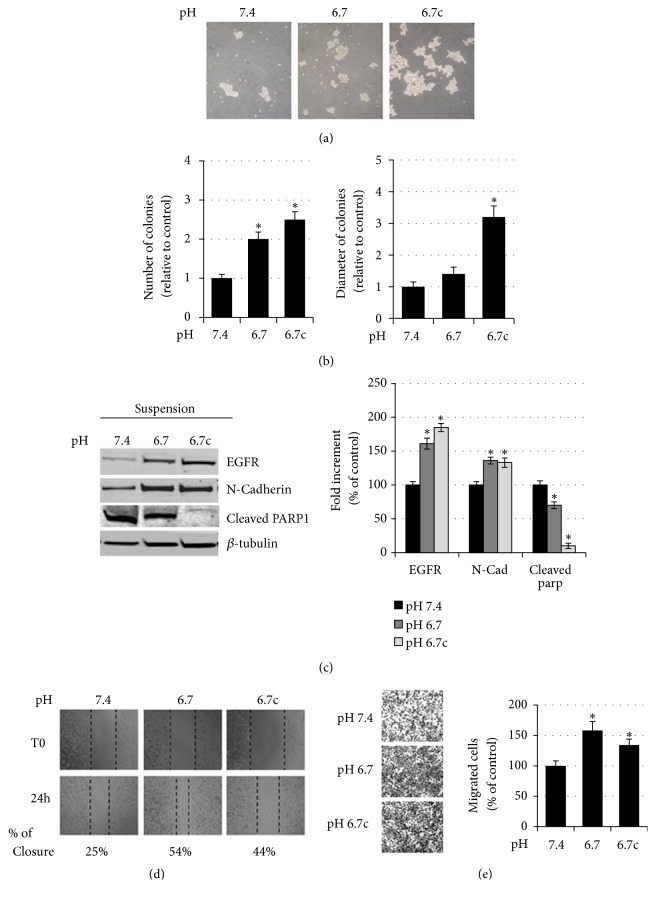
*Evaluation of the anoikis resistance of acidic melanoma cells grown in agarose*. (a) Representative images of acidic and nonacidic A375M6 melanoma cells allowed to growth for other 5 days on agarose-coated dishes and (b) quantification of the number and the dimension of relative colonies. (c) Representative images (left) of immunoblotting for EGFR, N-cadherin, cleaved PARP1, and *β*-tubulin of acidic and nonacidic A375M6 melanoma cells allowed to growth for other 5 days on agarose-coated dishes and (right) densitometry graph of protein expression. (d) Representative images of the wound healing assay on acidic and nonacidic A375M6 melanoma cells allowed to growth for other 5 days on agarose-coated dishes. Images are taken immediately after scratching (t0) and 24 hours later (24h). Percentage indicates wound closure compared to t0. (e) Representative images (left) of invasiveness of acidic and nonacidic A375M6 melanoma cells allowed to growth for other 5 days on agarose-coated dishes and quantitative analysis (right) of the number of cells that migrated through Matrigel. Each experiment was conducted in triplicate and data are expressed as mean ± SEM of at least three independent experiments. *∗* p<0.05.

To determine the effect of loss of adhesion on human melanoma cells grown under standard or low pH, we cultured cells on agarose-coated dishes. Agarose is often used as a support to prevent cell adhesion, mimicking survival, grown in suspension. Acidic and nonacidic cells were distributed in agarose-coated plates and grown for 5 days in complete medium. At that time, the number of tumor cell aggregates was evaluated, disclosing the higher ability of acidic melanoma cells, transient and chronic, to give rise to a higher number of cell aggregates (Figure 2(a)). In the case of chronic acidic melanoma cells, aggregates were also characterized by a more elevated diameter (Figure 2(b)). Melanoma cells collected from the aggregates were investigated for the level of expression of specific markers and their migration ability. As reported by NM Forfaria* et al*., anoikis-resistant melanoma cells are endowed of highly migratory and invasive properties [27].

We found that cells, from both transient and chronic cell aggregates, express an enhanced level of EGFR in addition to N-cadherin (Figure 2(c)) as we have demonstrated in parental cells [23]. When we tested the cleaved poly ADP-ribose polymerase 1 (PARP1), as a marker of apoptosis, chronic acidic melanoma cells recovered from agarose-coated dishes show a more apoptosis resistance phenotype than transient acidic cells and control cells. This last finding might support why aggregates of transient acidic cells are smaller than those of chronic acidic cells at the end of the experiments. The evaluation of amoeboid migration by scratch analysis, also known as wound healing assay (Figure 2(d)), and of the mesenchymal migration through Matrigel-coated filters of cells that survived in agarose coated dishes (Figure 2(e)), revealed that acidic cells recovering from aggregates also express a better migratory phenotype.

### 3.3. Anoikis Resistant Phenotype in Rocking Melanoma Cells

**Figure 3:**
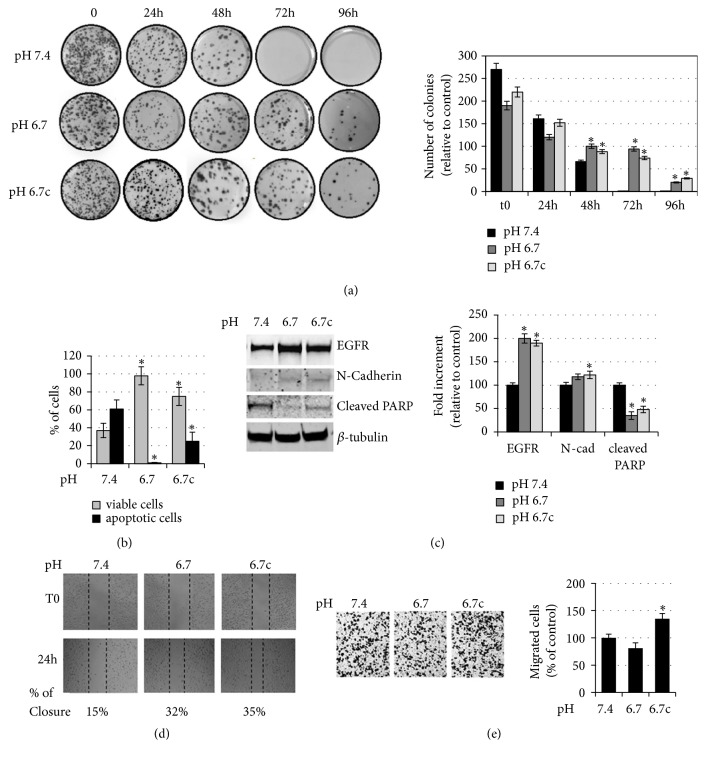
*Evaluation of the anoikis resistance of acidic melanoma cells grown in rocking conditions.* (a) Representative images (left) of clonogenic efficacy in acidic and nonacidic A375M6 melanoma cells after exposure to 24-96 h of rocking condition. After treatment, the cells were seeded in 100 mm plates (5000 cells/dishes, in triplicate) and incubated for 12–14 days. Quantification (right) of cloning efficiency expressed as number of colonies. (b) Annexin V/PI flow cytometric analysis of acidic and nonacidic A375M6 melanoma cells after exposure to 48h of rocking condition; the vital cells were negative for both annexin V and PI staining; the total cell death was the sum of cells positive for annexin V, PI, or both stainings. (c) Representative images (left) of immunoblotting for EGFR, N-cadherin, cleaved PARP, and *β*-tubulin of acidic and nonacidic A375M6 melanoma cells and after exposure to 48h of rocking condition and (right) densitometry graph of protein expression. (d) Representative images (left) of the wound healing assay on acidic and nonacidic A375M6 melanoma cells after exposure to 48h of rocking condition. Images are taken immediately after scratching (t0) and 24 hours later (24 h). Percentage indicates wound closure compared to t0. (e) Representative images (left) of invasiveness of acidic and nonacidic A375M6 melanoma cells after exposure to 48h of rocking condition and quantitative analysis (right) of the number of cells that migrated through Matrigel. Each experiment was conducted in triplicate and data are expressed as mean ± SEM of at least three independent experiments. *∗* p<0.05.

**Figure 4:**
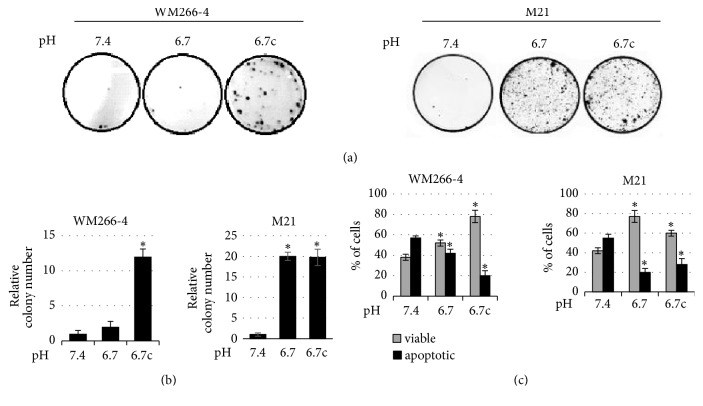
Evaluation of anoikis resistance of WM266-4 and M21 melanoma cells. (a) Representative images of clonogenic efficacy of acidic and nonacidic WM266-4 and M21 melanoma cells after exposure to 72h of rocking condition. After treatment, the cells were seeded in 100 mm plates (5000 cells/dishes, in triplicate) and incubated for 15 days. (b) Quantification of cloning efficiency expressed as number of colonies. (c) Annexin V/PI flow cytometric analysis of acidic and nonacidic WM266-4 and M21 melanoma cells after exposure to 48h of rocking condition. Each experiment was conducted in triplicate and data are expressed as mean ± SEM of at least three independent experiments. *∗* p<0.05.

To induce anoikis, cells were also prevented from adhering to the plastic of cultured flasks by a rocking procedure. Acidic and nonacidic adapted melanoma cells were suspended in media free of growth factors and placed in sterile nonadhesive 50 ml tubes that have been fixed on a Mini rocker platform shaker (20° angle) for 24-96 hours, at room temperature at a speed of 30 cycles/min. Under these conditions, the tubes were gently rocked and the constant movement of culture medium ensures a continuous suspension condition also preventing cell clumping. Cells which have been cultured under rocking were collected and their cloning efficiency was evaluated on standard culture condition. After 2 weeks of growth, as reported in Figure 3(a), we found that at the 48th hour of rocking a slight difference in the clonogenic ability between acidic cells and standard cells starts to be appreciated. Indeed, measurement of level of apoptosis, determined by the annexin method, indicates a significantly higher number of death cells in control melanoma cells (more than 55%) than in acidic cells (5-25%) after 48 hours of rocking condition (Figure 3(b)). In addition, acidic cells, either transient or chronic, were able to express a high capacity to give rise to numerous cell clones, while rocked melanoma cells grown in standard pH were unsuccessful (Figure 3(a)). These data indicate that acidic melanoma cells express a more resistant anoikis phenotype. In order to link anoikis resistance to specific markers and migratory ability, we collected 48-hour-rocking melanoma cells for the following experiments. Anoikis resistant cells derived from acidic melanoma cells conserved a slightly higher level of N-cadherin expression, consistent with a more EMT profile (Figure 3(c)). Associated with EMT profile, these latter melanoma cells are characterized by a faster ability to close the wounds (Figure 3(d)) as a significant higher ability to cross Matrigel-coated filters (Figure 3(e)). Regarding apoptotic escaping and cell survival, melanoma cells collected from acidic, transient and chronic, clones show a clear reduction of cleaved PARP1 and an enhanced expression of EGFR (Figure 3(c)). To verify whether the acidosis-induced anoikis-resistant phenotype observed in A375M6 cells is an aspect even of other melanoma cell lines grown in acidic microenvironment, we also undertook WM266-4 and M21 melanoma cells to rocking condition for 72h. As shown in Figures 4(a) and 4(b), both acidic WM266-4 (in particular chronic acidic cells) and M21 cells expressed a higher ability to give rise to cell colonies. Moreover, the determination of the level of apoptosis indicates a significantly lower number of death cells in acidic cells than in control cells at the 48th hour of rocking condition (Figure 4(c)).

**Figure 5:**
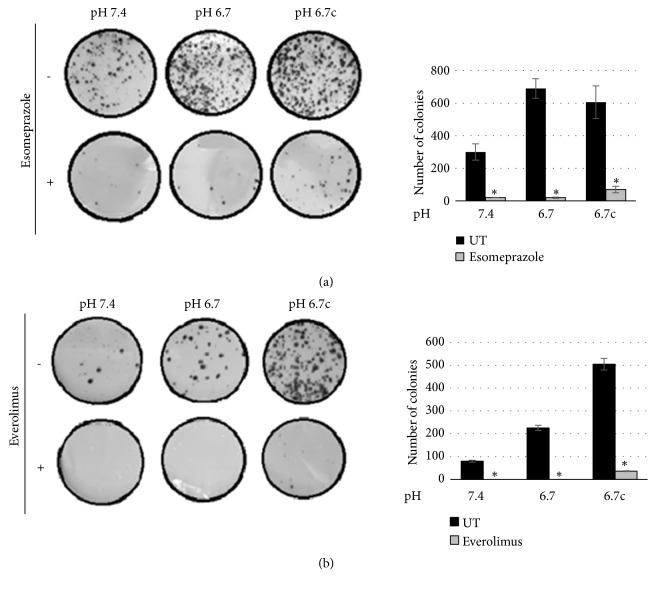
*Cloning efficiency of rocking melanoma cells exposed to esomeprazole or everolimus.* (a) Representative images (left) of clonogenic efficacy of acidic and nonacidic A375M6 melanoma cells after exposure to 48h of rocking condition in the presence or in the absence of 100 *μ*M esomeprazole (a proton-pump inhibitor). After treatment, the cells were seeded in 10 mm plates (5000 cells/dishes, in triplicate) and incubated for 15 days. Quantification (right) of cloning efficiency expressed as number of colonies (untreated, UT). (b) Representative images (left) of clonogenic efficacy of acidic and nonacidic A375M6 melanoma cells after exposure to 48h of rocking condition in the presence or in the absence of 2 *μ*M everolimus (an inhibitor of mTOR). After treatment, the cells were seeded in 100 mm plates (5000 cells/dishes, in triplicate) and incubated for 15 days. Quantification (right) of cloning efficiency expressed as number of colonies (untreated, UT). Data are expressed as mean ± SEM of at least three independent experiments. *∗* p<0.05.

Considering our previous experience on the ability of esomeprazole, a proton pump inhibitor that requires acidosis for its activation, to prevent NF-*κ*B activation [17, 28], we rocked for 48 hours acidic and nonacidic melanoma cells in the presence of esomeprazole.  Figure 5(a) shows the ability of esomeprazole to abrogate cloning efficiency of acidic and nonacidic anoikis resistant melanoma cells. In addition, considering the fact that low pH microenvironment promotes in melanoma cells a vemurafenib resistant phenotype, which could be targeted using an mTOR inhibitor, such as everolimus [23], we decided to test sensitivity of anoikis resistant acidic and nonacidic melanoma cells to everolimus. Indeed, as shown in Figure 5(b), everolimus is effective in abrogating cloning efficiency of acidic melanoma cells rocked for 48 hours and also of melanoma cells grown in standard pH.

Overall, a low pH adaptation, both transient and chronic, exerts an anoikis resistant phenotype in melanoma cells endowed with increased proliferative signals, reduced apoptosis markers, and an esomeprazole/everolimus sensitivity.

### 3.4. In vivo Evaluation of Anoikis-Resistant Phenotype

**Figure 6:**
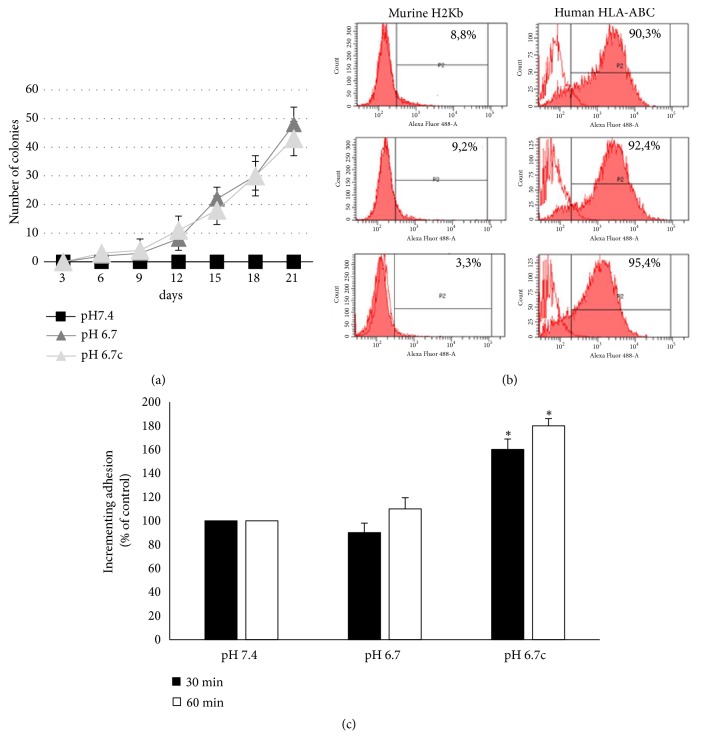
*In vivo evaluation of anoikis resistance of acidic melanoma cells.* (a) Emerging colonies derived from blood sample obtained from mice (n=3) i.v. injected with acidic and nonacidic A375M6 melanoma cells; colonies with over 20 cells were counted. (b) Representative images of flow cytometer analysis of HLA-ABC or H2Kb antigen expression in pooled cell population that recovered from blood samples after 21 days. (c) Evaluation of adhesiveness of acidic and nonacidic A375M6 melanoma cells toward activated endothelial cells at 30 and 60 min through flow cytometer analysis of acidic and nonacidic CSFE-positive A375M6 melanoma cells and activated endothelial cells (CSFE-negative) cocultures. Images are representative of experiments performed in triplicate; data are expressed as mean ± SEM of at least three independent experiments. *∗* p<0.05.

We have next investigated the ability of melanoma cells to survive in the blood stream. To test this phenomenon, an equal number of melanoma cells (1x10^6^ cells) adapted to an acidic medium, either transiently or chronically, and melanoma cells grown under standard conditions (pH 7.4) were inoculated intravenously into immune deficient mice. Mice were sacrificed 15 min after tumor cells injection to collect, by a cardiac puncture, 0.5 ml of blood. A period of 15 min represents a time of observation long enough to evaluate resistance of tumor cells in blood stream and, at same time, short enough to prevent trapping of melanoma cells in the capillary bed of the lungs [29]. Blood of injected mice was treated with a hypotonic medium to eliminate red blood cells; thereafter the heterogeneous cell suspension was distributed in culture dishes. At the 21^st^ day of growth, we counted number of colonies and, as we report in Figure 6(a), control cells give no colonies, whereas both types of acidic melanoma cells reach a range of 40-50 colonies. To validate these experiments, melanoma cells were collected from colonies and tested, by immunofluorescence, for level of expression of murine and human Major Histocompatibility Complex (MHC) class I antigens.  Figure 6(b) shows that cells collected from the clones express human MHC class I antigens (A, B, and C alleles), but not murine H2-Kb antigen. Thus, acidic cells express an enhanced ability to survive in blood stream. A stringent request for perfect “circulating tumor cells” (CTC) is the capacity to adhere to endothelial cells, a step before extravasation and secondary organ colonization. Therefore, we compared the adhesive properties of acidic cells with that of control cells. Labeled melanoma cells were added to a monolayer of human endothelial cells and incubated for 30-60 min at 37°C. Then, nonadherent cells were washed out using a phosphate buffer, and both the endothelial cells and the corresponding attached melanoma cells were collected by trypsinization. Flow cytometry analyses reported in Figure 6(c) reveal that chronic acidic melanoma cells show an enhanced aptitude to stick to endothelial cells. This finding deserves further investigations in order to clarify whether acidic melanoma cells are also endowed in metastatic niche formation, the final ability of a “perfect” CTC.

## 4. Discussion

Clarifying the mechanisms regulating the acquisition of a resistant anoikis program is necessary to develop new strategies to combat metastatic dissemination. Several strategic pathways may confer anoikis resistance to cancer cells, including activation of prosurvival pathways (e.g., PI3-K, MEK/ERK, and NF-kB), change in integrin expression, secretion of reactive oxygen species (ROS), and acquisition of an EMT profile [6, 7].

Here, we show for the first time that an acidic pH medium, related to Warburg effect and/or anaerobic glycolysis of tumor cells, promotes in human melanoma cells an anoikis resistant phenotype, a further trait of malignancy. Anoikis resistance is useful to survive in blood stream and in the various circumstances in which melanoma cells endowed with an aggressive phenotype need to detach from extracellular matrix (ECM) and survive in suspension. Indeed, melanocytes are kept in their tissue compartment through integrin/ECM interactions, but the underlying dermis is rich in collagen, failing to support melanocyte adhesion and survival, as they do not express suitable integrins. However, during melanoma dermis invasion, the upregulation of *α*v*β*3 integrin allows them to receive antiapoptotic signals to survive anoikis [30]. Our model of acidic cells was represented by transient (24 hours) and chronic (three months)-adapted murine sarcoma viral oncogene homolog B (BRAF) melanoma cells to mimic short and long exposure of cancer cells to low pH. pH 6.7 is the level of acidity more frequently observed in spontaneous human melanoma. During our investigations on acidity and melanoma progression we collected several findings suggesting that acidic melanoma cells could be a very good candidate for also expressing an anoikis resistance, such as a switch to an EMT profile (high N-Cadherin, low E-Cadherin), high MMP-dependent invasive ability, apoptotic resistance, and an AKT/mTOR privileged pathway [20, 23]. Indeed many recent studies show that EMT increases metastatic potential of cancer cells by mediating anoikis resistance [31, 32]; particularly it was demonstrated that the depletion of E-cadherin promotes mammary cell survival following loss of cell adhesion to the ECM [33], and N-cadherin expression protects melanoma cells from anoikis [34, 35] and confers apoptosis resistance to esophageal carcinoma cells [36]. In addition to an increased expression of N-Cadherin, acidic cells showed an upregulation of *α*v*β*3 and *α*v*β*5 integrins and ERK and AKT activation. Indeed, to alter the integrin repertoire in response to selective microenvironmental pressures is considered another strategy to avoid anoikis [37] and a key player in integrin‐mediated signal transduction leading to anoikis protection is ERK, whereas Akt is an essential element of cell survival signaling as integrin‐, growth factor‐, and cell–cell anchorage‐mediated signals converge to its activation to grant survival of cells [38].

Another important observation obtained previously in our laboratory was that when acidic melanoma cells were injected intravenously with nonacidic cells, they improved the capability of cells grown at standard pH to give rise to lung metastasis [20]; this fact suggested that the nonacidic melanoma subpopulation was able to promote the development of lung colonies and that the prometastatic role of acidic cells could be the protection of nonacidic cells in blood stream and/or the ability to favor their attachment on lung endothelial cells and the following lodgment into the lung parenchyma. Along these suggestions, we validated that acidic melanoma cells are endowed with an anoikis resistant phenotype growing both acidic and control cells in nonadherence conditions, for example, grown on agarose-coated dishes, under rocking, and finally exposing cancer cells to the* in vivo* blood stream. We were able to demonstrate that acidic melanoma cells give a more elevated number of cell aggregates when they were plated on agarose-coated dishes. At the same time, acidic melanoma cells exposed to a continuous rocking expressed a better clonogenic ability than control cells, for example, the capacity to develop a very high number of clones. Thus, the acquisition of a low pH phenotype, after transient but also chronic exposure to an acidic medium, implies the anoikis resistance. It is possible that acidity generates an anoikis resistant profile by modulating EMT program, expression of adhesion molecules (specific *α*v integrins), growth factor receptor (EGFR), and specific transcription factor pathways (pAKT/mTOR, and NF-kB). Thus, a molecular signature elicited by a reduced extracellular pH is adequate to confer the ability to survive without attachment to the ECM.

An important distinguishing factor of anoikis resistant acidic melanoma cells was the upregulation of EGFR and AKT pathway. Epidermal growth factor and its receptor can lead to increased PI3-K signaling. This signaling has an effect on downstream substrate AKT and its phosphorylation. This activates the mTOR (Target of Rapamycin) which contributes to the development of an anoikis resistance. Boisvert-Adamo K and Aplin AE showed that two major survival pathways work in BRAF melanoma cells to provide a protection from anoikis: the B-RAF-MEK and the PI-3 kinase-AKT signaling [39]. Indeed, A375-M6 melanoma cells are BRAF mutated (V600E) and when they are adapted to an acidic medium they acquire an enhanced activation of PI-3 kinase-AKT pathway crucial for vemurafenib resistance development, as we have previously demonstrated [23]. Indeed, we have demonstrated that an mTOR/NF-kB inhibitor drives a significant killing of acidic anoikis resistant melanoma cells. Also, the subpopulation of nonacidic anoikis resistant cells was sensitive to mTOR/NF-kB inhibition.

PI3 kinase-AKT and ERK activation is reported to characterize melanoma cells upon anchorage independency and syndecan 2 (SDC2) increased expression. SDC2 is also associated with a higher expression of migratory and melanin synthesis [40].

Using RNA interference, Boisvert-Adamo K.* et al*. showed that depletion of myeloid cell leukemia (Mcl)-1 renders mutant B-RAF melanoma cells sensitive to anoikis. By contrast, minor effects were observed following depletion of either Bcl-2 or Bcl-XL. Mcl-1 expression is enhanced in melanoma cells compared with melanocytes and upregulated by the B-RAF-MEK-extracellular signal-regulated kinase pathway through the control of Mcl-1 protein turnover [41]. Among the other markers, the signal transducer and activator of transcription (STAT)3, that is associated with anoikis resistance in melanoma cells, as Fofaria and Srivastava demonstrated [27], was unmodified by low pH exposure (data not shown).

Wound healing and Boyden's chamber assays suggested that the acidic anoikis-resistant melanoma cells express an enhanced motility and invasiveness through Matrigel, according to the recent observation that anoikis resistant cells show increased cell migration and invasion [42]. Finally, the ability of acidic melanoma cells to survive in blood stream is particularly interesting because this finding not only sustains* in vivo* the anoikis profile of these cells but also predicts the possible involvement of acidic-adapted melanoma cells in the following steps of metastatic cascade, such as endothelial cell adhesion, extravasation, and lodgment into the secondary organ. We may consider these final steps as candidates for our next research effort.

## 5. Conclusions

In summary, our findings show that a low pH microenvironment is crucial in conferring to melanoma cells an anoikis resistant trait. This evidence underscores the importance of targeting low pH of melanoma, likely combining mTOR/NF-kB inhibitors.

## Data Availability

The data used to support the findings of this study are included within the article.

## Abbreviations

mTOR: Mammalian target of rapamycin
NF-kB: Nuclear factor kappa-light-chain-enhancer of activated B cells
CTC: Circulating tumor cell
ECM: Extracellular matrix
Bcl: B-cell lymphoma
HIF: Hypoxia-inducible factor
LDH: Lactate dehydrogenase
MCT: Monocarboxylate transporter
pHe: Extracellular pH
EMT: Epithelial-to-mesenchymal
AKT: Protein-chinasi B o PKB
ERK: Extracellular-signal-regulated kinase
EGFR: Epidermal growth factor receptor
Tie: Tyrosine kinase with immunoglobulin-like and EGF-like domains
MEK: Mitogen-activated protein kinase
PARP: ADP-ribose polymerase
MHC: Human Major Histocompatibility Complex
PI3K: Phosphatidylinositol 3-kinase
ROS: Reactive oxygen species
BRAF: Murine sarcoma viral oncogene homolog B
uPAR: Urokinase-type plasminogen activator receptor
SDC: Syndecan
Mcl: Myeloid cell leukemia
STAT: Signal transducer and activator of transcription.
